# Book Review

**Published:** 1908-12

**Authors:** 


**Light and X-ray Treatment of Skin Diseases.** By Malcolm Morris and S. Ernest Dore, M.D. Pp. x., 172. London: Cassell and Company, Limited. 1907.—This work is a useful addition to Cassell's *Modern Methods of Treatment* Series. It is the first attempt of which we are aware to review dispassionately the new physical methods of treatment in dermatology. All explanation of the physics and electrics involved in the production of the various radiant phenomena are excluded. Description of apparatus is also left out. This limits the work to the dermatology of the subject, and is, therefore, of greater value to practitioners, nurses, and all who wish to know succinctly the scope of a new series of therapeutics. The authors were among the earliest to use the light lamps in the treatment of skin diseases in England. They have shown judgment in assessing their own results, and have altogether approached the subject in a most unbiased way. It might perhaps be said that in some respects they are too pessimistic, though this is preferable to an undue optimism. There is a little uncertainty in some of the cases as to what method of treatment was relied upon most, but this does not greatly matter where the choice of method is largely determined by the nature of the case itself and the choice of the operator. The book should prove a welcome and useful addition to the literature of the subject.

**What to do in Cases of Poisoning.** By William Murrell, M.D. Tenth Edition. Pp. 280. London: H. K. Lewis. 1907. —A book which has reached its tenth edition hardly needs a review. This one, however has undergone such thorough revision, and has had so many new articles added to it, that it may be well to call attention to the fact that it contains directions for the treatment of poisoning by recently discovered substances such as dionin, stovaine, veronal, ethidene dichloride. The author playfully requests intending suicides, who contemplate employing still more unusual combinations, to first communicate with him, that the book may be kept fully up to date. This scientific enthusiasm has not equally affected his proof reader, who shows signs of breathless haste. Thus we notice the curious statement that rectified spirit contains 90 per cent. of water (p. 57), laburnum is spelt laburnam, mercurial erethism is given as erithism (p. 264).

Perhaps these spellings are intended for a future edition. The applicant for admission to a retreat is told to bring a certificate of "immoral" character attested by two justices, whereas the Act specifies "inebriety" alone. Such a direction might cause needless disappointment to a hopeful candidate under the present state of the law, though some moralists hold that the two terms are identical.

**Index-Catalogue of the Library of the Surgeon General's Office, United States Army.** Second Series. Vol. XII. O—Periodicals. Pp. 978. Washington: Government Printing Office. 1907.—Only those who have to inquire into the literature of any particular subject can fully appreciate the great value of this monument of painstaking classification of the contents of a library containing 160,412 bound volumes and 282,994 pamphlets. The present volume includes 5,476 author-titles, 10,996 subject-titles of separate books and pamphlets, and 35,324 titles of articles in periodicals. It is only by quoting such figures that we can give to the uninitiated any conception of the extent and usefulness of the work.

**Chart.** Dr. HEBBLETHWAITE. London: The Scientific Press Ltd.—Of the making of charts there is no end. This is a new one, to be filled in with ink, and red and blue pencils for temperatures, pulse and respirations. The chart is arranged for any intervals of time, and vertical lines should be drawn at the end of each day across the blank spaces allotted to date, hour, day of disease, &c.

**Wellcome's Photographic Exposure Record and Diary.** 1908.—In addition to the exposure record and diary, which seem most excellently arranged, this pocket-book contains practically all the information necessary about the usual photographic procedures—developing, printing, &c.—for the beginner, and for the matter of that for the more advanced photographer. We notice that the fashionable "time factor" method of development is described, and the time factors for the various "tabloid" developers given. In addition there is a good deal of useful information given about weights and measures, temperatures, &c. The exposure calculator at the end is alone worth the price of this little photographic pocket-book.

**Life Insurance and General Practice.** By E. M. BROCKBANK, M.D. Pp. xiv., 288. London: Henry Frowde and Hodder and Stoughton. 1908.—The first half of this book describes the routine method of making medical examinations for life insurance. The author hopes that his method will be of assistance to those who make similar examinations for the first time. The second part of the book, on "impaired lives," is, we venture to think, of much more value, inasmuch as all the serious problems in life insurance work are incurred with this class of cases, in which there is scope for much individual skill in diagnosis and prognosis.

The experience of the author, and the views he has gathered from various sources, cannot fail to be interesting and instructive to those who take any interest in the work of life insurance. It must, however, be remembered that no examiner can do more than give his opinion on the present condition of the applicant. Any prognosis of future possibilities is beset with so many fallacies, that it is only fair that the applicant for insurance should have the benefit of whatever doubts may exist.

**Manual of Bacteriology.** By ROBERT MUIR, M.D., and JAMES RITCHIE, M.D. Fourth Edition. Pp. xxi., 605. Edinburgh: Young J. Pentland. 1907.—In this edition of their widely-used manual the authors have incorporated the results of the research work of the last few years, in addition to a recital of some of the more reasonable hypotheses. Fortunately, however, they have kept the book within the limit of the student's purse, and, presumably, of his capabilities. Syphilis, relapsing fever, and trypanosomiasis are treated at greater length than before. A number of new methods of cultivation and staining are also included. There is no doubt that this manual must be regarded as the best investment a student can make when he commences the study of bacteriology.

**Aids to Pathology.** By HARRY CAMPBELL, M.D. Pp. viii., 176. London: Baillière, Tindall & Cox. 1908.—It is obvious that a little book of 176 small pages cannot pretend to give an exhaustive account of the known facts of pathology. As a framework, on which the student can build up with details acquired from the *post-mortem* room, the museum, and the laboratory, it must serve a very useful purpose, and it is to be expected that the section on immunity and opsonins will help greatly to elucidate a somewhat difficult problem in serum therapeutics.

**Disease, its cause and prevention.** By G. E. RICHMOND, M.D. London: H. K. Lewis. 1907.—The main object of this essay is to point out the very large number of diseases which are either spread by food, or directly due to impurities found in articles of diet or articles in common use. If disease is preventable, then it ought to be prevented. To this end medical practitioners should be friends, and not rivals; they should strive for health, and not for patients; their services should be adequately and generously remunerated. To these safe views we can only say, "Quite so."

**Diets in Tuberculosis: Principles and Economics.** By NOEL DEAN BARDSWELL, M.D., and JOHN ELLIS CHAPMAN. Pp. viii., 184. London: Henry Frowde and Hodder and Stroughton. 1908.—It is difficult to estimate the far-reaching nature of the sanatorium movement; certainly as a means of cure, of prevention and of education it has been an immense power for good, both for the consumptive and for the general welfare of the community. The principles are: the carefully-ordered life, with freedom from fatigue and worry; carefully-regulated hours of rest and exercise; and, most important of all, abundance of fresh air and generous, but not excessive, feeding. This book shows that these principles are within reach of every class of consumptive.

**Protozoa and Disease.** By J. J. JACKSON CLARKE, M.B. Part II. Pp. xii., 138. London: Baillière, Tindall & Cox. 1908. —In this interesting little book the author strongly advocates the protozoal origin of various diseases, notably small-pox, syphilis and sarcoma. He first refers to the relationship between the spirochæta pallida and syphilis, and also to the better established one between the trypanosome Gambiense and sleeping sickness. Starting on this ground, he endeavours to prove that various appearances noted in sections of sarcomata are due to a parasite of the protozoan group. His arguments are based largely on analogy, and his attempts to support them by the microscopical appearances are by no means convincing or conclusive. In no case has he been able to trace the life history of the supposed parasite, and it must be confessed that the appearances noted by him in sarcoma cells are suggestive of degenerative changes in the nuclei and protoplasm of cells rather than of parasites. The theories advanced are interesting, but entirely lacking in proof.

**Stray Leaves and Some Fruit on Cancer and Tuberculosis.** By HENRY D. MCCULLOCH, M.B. Pp. 49. London: John Bale, Sons, & Daniellson, Ltd. 1907.—This is one of the many speculative treatises on abstruse medical subjects in which certain philosophic but unpractical men delight. The stray leaves one can see, but the fruit is not so obvious. On the whole, one may say that no *a priori* reasoning, however inspired, and no careful consideration of other men's work are likely to be of any avail in such fields unless combined with original investigation. Therefore, we can only recommend this work as an incentive to thought, and because of the various quotations it contains; and the incentives are on familiar lines. The book is carefully written. One of the most interesting features is a Sanskrit quotation on the cover, and an ingenious interpretation of the same at the end.

**Diseases of the Eye.** By J. HERBERT PARSONS, D.Sc., M.B., B.S. Pp. x., 664. London: J. & A. Churchill. 1907.—This book has been written as a manual for students and practitioners. It does not attempt the scientific detail that the author could well have given out of the stores of his knowledge acquired in the laboratory or from wide reading; still, it is based on good scientific lines, and accurately and clearly describes what is essential for a sound practical knowledge of the various affections of the eyeball and of the function of vision. The author does not claim to have dealt exhaustively with the whole range of ophthalmology, but he appears to have omitted nothing that is essential for his intended readers. The anatomy of the structures of the eye is well dealt with, especially, perhaps, the neurology of vision and the nerve control of the ocular muscles. Refraction is fully considered from a practical standpoint, founded on a groundwork of physics and of physiology. Disorders in the mobility of the eyes is a difficult subject, but it is admirably handled. We know of no description in so limited a space that gives a better arranged or more scientific consideration of the subject of strabismus, diplopia and latent defects in the position of the eyes. The various diseases of the eye are sufficiently described, and the pathological conditions are very thoroughly considered, having regard to the space at the author's disposal. There are many good text-books on ophthalmology, but we think Mr. Parsons has been more than justified in adding to the list. We recommend his manual confidently to anyone who wishes to study diseases of the eye.

**Ilford X-ray Plates.**—Ilford Company, Ilford.—We have received a sample of these plates, which we have tested, and find them excellent in every way. They are rapid in action, and give good definitions and density. We are very glad to find that at length English manufacturers are thinking it worth their while to cater for the wants of the radiographer, as up to the present the only plates which were superior to others for X-ray work were of Continental manufacture. The Ilford X-ray plates seem, as far as we can judge from the few exposures we have made, to be at any rate equal to, if not superior, to any plate in the market, and we should think they have only to become known to be very extensively used.

**Hygiene and Public Health.** By LOUIS C. PARKES, M.D., D.P.H., and HENRY R. KENWOOD, M.B., D.P.H. Third edition. Pp. xi., 620. London: H. K. Lewis. 1907.—This text-book has now been established for some years as a sound and reliable guide to the general study of public health. The third edition has been carefully brought up to date, and may be safely recommended to the student for the diploma examinations. The type is clear, and the slight increase in size of the page improves the general appearance of the book.

**The Experimental Prophylaxis of Syphilis.** By PAUL MAISONNEUVE. Translated, and with an introduction, by FERNAND L. DE VERTEUIL, M.B. Bristol: John Wright & Sons, Ltd. 1908.—This little brochure of about 100 pages gives an account of the work of Metchnikoff and Roux in connection with the ætiology and prophylaxis of syphilis. The author is descriptive rather than critical, but gives a readable account of the subject. The practitioner must not accept all the statements unhesitatingly. Of course much that is written may be applied to every-day cases, but the present state of our knowledge on the subject does not permit of dogmatism, and at the time of writing this review some of the data require alteration. It is a significant fact that the extent of the syphilis produced varies distinctly with the amount of the virus inoculated, and we cannot, therefore, hope to apply vaccination therapy to this disease. Excision of the infected area is the best practice, but this is not often possible.

**Diseases of the Larynx.** By HAROLD BARWELL, M.B. Pp. xii., 266. London: Oxford Medical Publications.—Within the small space comprised in this manual, the utmost that could be done satisfactorily is to afford the student and practitioner a brief description of laryngoscopy and the various affections of the larynx. This the author has done admirably, the illustrations are clear and useful, the text sound, practical and well-balanced, and the directions for treatment just the kind that the general practitioner needs for guidance and help in cases of difficulty coming within the scope of his daily work.

**Physical Methods in the Treatment of Heart Disease.** By ARTHUR G. DAMPIER-BENNETT. Pp. 111. Bristol: John Wright & Sons, Ltd. 1907.—The author of this volume gives a very clear description of the various methods which may, in some cases, be successfully employed in the treatment of cardiac cases. These procedures include treatments by baths, rest, active and passive exercises, Schott movements, massage and electrical applications. A useful chapter is devoted to diet, in which not only are the main principles of the treatment by diet discussed, but also the mode of preparing some of the articles described in detail. Throughout the book stress is laid on the type of case likely to derive benefit from any given line of treatment, while the help which may also be obtained by drugs receives due prominence in the last chapter. Here we are surprised to read that the author recommends the smoking of opium under certain circumstances, a therapeutic method it might be dangerous to introduce.

**A Synopsis of Surgery.** By ERNEST W. HEY GROVES, M.D., M.S., B.Sc. Lond., F.R.C.S. Eng. Pp. viii., 486. Bristol: John Wright & Sons Ltd. 1908.—This epitome of the salient facts in surgical practice may be looked upon as a concentrated extract of surgery for hasty reference and revision. It needs, however, to be diluted largely before it can be digested or assimilated. The matter is arranged in forty-nine chapters, somewhat after the system of "Rose and Carless;" the headings, type and indented margins conduce to easy reference, for which the book is intended; it appears to be very carefully compiled, and forms a handy companion for the student and busy practitioner who has little time for anything but concentrated pabulum. The work has been done by a master of his subject, and is thoroughly reliable.

